# Genome Sequence of Type Strain *Fonsecaea multimorphosa* CBS 980.96^T^, a Causal Agent of Feline Cerebral Phaeohyphomycosis

**DOI:** 10.1128/genomeA.01666-16

**Published:** 2017-02-16

**Authors:** Aniele C. Ribas Leao, Vinicius Almir Weiss, Vania Aparecida Vicente, Flavia Costa, Amanda Bombassaro, Roberto Tadeu Raittz, Maria Berenice R. Steffens, Fabio Oliveira Pedrosa, Renata R. Gomes, Valter Baura, Helisson Faoro, Michelle Zibetti Tadra Sfeir, Eduardo Balsanelli, Leandro F. Moreno, M. Javad Najafzadeh, Sybren de Hoog, Emanuel Maltempi Souza

**Affiliations:** a Laboratory of Bioinformatics, Professional and Technological Education Sector, Federal University of Paraná, Curitiba, Paraná, Brazil; b Department of Biochemistry and Molecular Biology, Federal University of Paraná, Curitiba, Paraná, Brazil; c Department of Bioprocess Engineering and Biotechnology, Federal University of Paraná, Curitiba, Paraná, Brazil; d Laboratory of Microbiology, Department of Pathology, Federal University of Paraná, Curitiba, Paraná, Brazil; e Instituto Carlos Chagas, Fundação Oswaldo Cruz, FIOCRUZ, Curitiba, Paraná, Brazil; f CBS-KNAW Fungal Biodiversity Centre, Utrecht, the Netherlands; g Department of Parasitology and Mycology, School of Medicine, Mashhad University of Medical Sciences, Mashhad, Iran

## Abstract

A draft genome sequence of type strain *Fonsecaea multimorphosa* CBS 980.96^T^ was obtained. This species was first isolated from a cat with cerebral phaeohyphomycosis in Queensland, Australia.

## GENOME ANNOUNCEMENT

Pathogenic species of the *Herpotrichiellaceae* are able to cause mutilating skin infections known as chromoblastomycosis as well as disseminated phaeohyphomycosis, and a few species such as *Rhinocladiella mackenziei* and *Cladophialophora bantiana* can cause cerebral phaeohyphomycosis (1, 2). Clinical symptoms commonly found in patients with cerebral phaeohyphomycosis are severe headache, seizures, paralysis of one or more oculomotor muscles, and weight loss, leading to death (3). Once the fungus reaches the nervous system, the excessive production of melanin leads to inflammation and necrosis of brain tissue. Cerebral phaeohyphomycosis caused by *Fonsecaea* species are rare (4), but *Fonsecaea monophora* has been repeatedly isolated from humans (5) and *F. pugnacious* was isolated only once (6). *F. multimorphosa* CBS 980.96^T^ was isolated from an abscess in the left occipital lobe of the cerebrum of a female cat in Queensland, Australia (3). The feline was 18 months of age and had dilated pupils with no reflection, enlarged liver, inflamed bile duct, and congested lungs, with no cutaneous and/or subcutaneous lesions (3).

*F. multimorphosa* CBS 980.96^T^ was grown on Sabouraud’s glucose agar (SGA) at 28°C for 7 days. DNA was extracted using the CTAB (cetyltrimethylammonium bromide) method and phenol-chloroform/isoamyl alcohol. A microbial DNA UltraClean kit was used for purification of total DNA. A DNA library for sequencing was prepared using the Nextera kit (Illumina) following the manufacture’s recommendations, and sequenced on a MiSeq sequencer using the paired-end (2 × 250 bp) sequencing method. A quality check of the sequenced reads was done using FastQC (7). SPAdes version 3.6.2 (8) was used for *de novo* assembly. Gap closure was performed using FGAP (9) and assembly coverage was determined using Bowtie2 (10). Gene prediction was performed using GeneMarkES version 4.29 (11). Annotation of the protein coding genes was based on best-hit searches against the NR database using RAFTS3 (12) and InterProScan (13). Aragorn version 1.2.36 (14) was used for tRNA gene identification.

A total of 7,445,102 paired-end reads were obtained for *F. multimorphosa* CBS 980.96^T^ genome sequencing. The reads were assembled into 163 scaffolds with a G+C content of 52.64% and an *N*_50_ of 392,992 bp. The genome size was estimated to be 33.4 Mbp. More than 97% of the reads were used for assembly yielding a genome coverage of 54×. A total of 11,772 protein-coding genes with at least one conserved domain were predicted and 32 tRNAs. The genes were also annotated based on a search of similarity with known proteins of other fungal species.

The genome sequence of *F. multimorphosa* CBS 980.96^T^ will be important for a better understanding of the basic mechanisms of adaptation to hostile environments, pathogenicity, and virulence of this organism.

### Accession number(s).

The whole-genome shotgun project of *F. multimorphosa* CBS 980.96^T^ has been deposited at DDBJ/EMBL/GenBank under the accession number LVCI00000000.
